# Integrins in Health and Disease—Suitable Targets for Treatment?

**DOI:** 10.3390/cells13030212

**Published:** 2024-01-23

**Authors:** Tanja Klaus, Christoph Hieber, Matthias Bros, Stephan Grabbe

**Affiliations:** Department of Dermatology, University Medical Center of the Johannes Gutenberg-University Mainz, Langenbeckstraße 1, 55131 Mainz, Germany; tklaus@uni-mainz.de (T.K.); chieber@uni-mainz.de (C.H.); mbros@uni-mainz.de (M.B.)

**Keywords:** αβ integrins, leukocyte receptors, β_2_ integrins, LFA-1, MAC-1, LAD-1, migration, phagocytosis, NETosis, reactive oxygen species

## Abstract

Integrin receptors are heterodimeric surface receptors that play multiple roles regarding cell–cell communication, signaling, and migration. The four members of the β_2_ integrin subfamily are composed of an alternative α (CD11a–d) subunit, which determines the specific receptor properties, and a constant β (CD18) subunit. This review aims to present insight into the multiple immunological roles of integrin receptors, with a focus on β_2_ integrins that are specifically expressed by leukocytes. The pathophysiological role of β_2_ integrins is confirmed by the drastic phenotype of patients suffering from leukocyte adhesion deficiencies, most often resulting in severe recurrent infections and, at the same time, a predisposition for autoimmune diseases. So far, studies on the role of β_2_ integrins in vivo employed mice with a constitutive knockout of all β_2_ integrins or either family member, respectively, which complicated the differentiation between the direct and indirect effects of β_2_ integrin deficiency for distinct cell types. The recent generation and characterization of transgenic mice with a cell-type-specific knockdown of β_2_ integrins by our group has enabled the dissection of cell-specific roles of β_2_ integrins. Further, integrin receptors have been recognized as target receptors for the treatment of inflammatory diseases as well as tumor therapy. However, whereas both agonistic and antagonistic agents yielded beneficial effects in animal models, the success of clinical trials was limited in most cases and was associated with unwanted side effects. This unfavorable outcome is most probably related to the systemic effects of the used compounds on all leukocytes, thereby emphasizing the need to develop formulations that target distinct types of leukocytes to modulate β_2_ integrin activity for therapeutic applications.

## 1. Introduction

The αβ integrin receptor family is a large group of heterodimeric surface receptors that bind various cell surface receptors and components of the extracellular matrix (ECM) to support cell–cell interaction and migration and are involved in cell differentiation and signaling processes [1]. Within this family, the β_2_ integrin subfamily is characterized by leukocyte-restricted expression [2], and its four family members exert multiple regulatory functions within the innate and adaptive immune systems [3,4].

**Figure 1:**
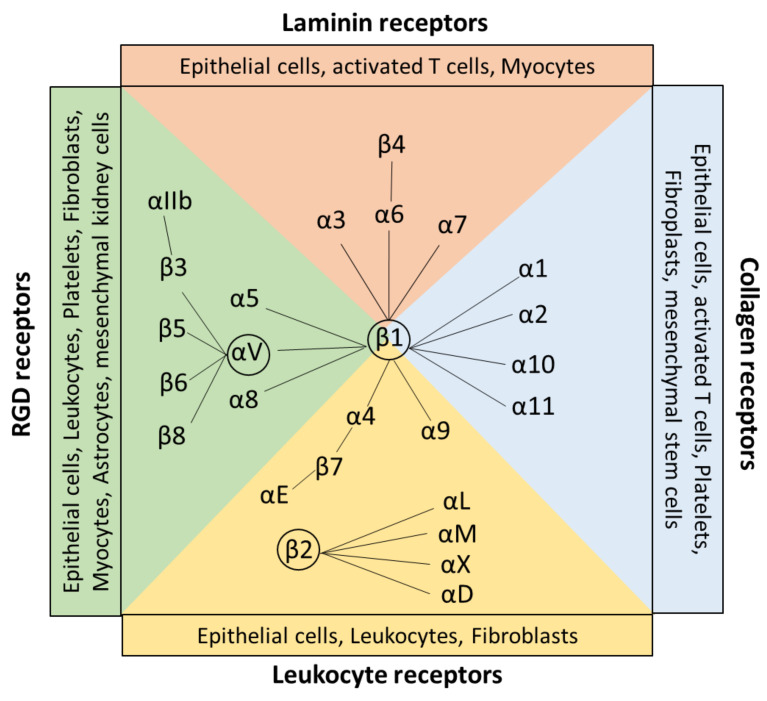
Classification and expression pattern of the αβ integrin receptor family. Integrin heterodimers consist of different combinations of the α and β subunits. In general, integrins are classified by their ligand specificity in RGD-recognizing integrins (α_5_β_1_, α_V_β_1_, α_V_β_3_, α_V_β_5_, α_V_β_6_, α_V_β_8_, and α_llb_β_3_) shown in green, laminin-binding integrins (α_3_β_1_, α_6_β_1_, α_7_β_1_, and α_6_β_4_) shown in red, collagen-binding integrins (α_1_β_1_, α_2_β_1_, α_10_β_1_, and α_11_β_1_) shown in blue, and leukocyte integrins (α_9_β_1_, α_4_β_1_, α_E_β_7_, α_L_β_2_, α_M_β_2_, α_X_β_2_, and α_D_β_2_) shown in yellow. The β_1_, β_2,_ and αV containing integrins built the largest groups of the family. All integrins are expressed by a wide range of cells, including, for example, leukocytes, epithelial cells, and fibroblasts. Created with BioRender.com.

Integrins are a large family of evolutionarily conserved heterodimeric surface receptors that are composed of an α and a β chain [1]. Integrin αβ heterodimers are divided into four classes based on ligand specificity and evolutionary origin, namely leukocyte, collagen-binding, Arg-Gly-Asp (RGD)-binding, and laminin-binding integrins (Figure 1) [5]. In vertebrates, 18 different integrin α subunits and 8 β subunits can be expressed [1,6,7], whereas β_1_, β_2_, and αV-containing integrins constitute the largest three groups of this family [6,8]. To date, in total, 24 β integrin combinations are known to be expressed by humans [5].

Usually, the ligand binding ability of αβ integrins is conferred by recognizing small peptide sequences of the ECM and both cell surface receptors and serum components [9]. Once they have bound to a target peptide, integrins provide adhesion and initial signaling, allowing cells to respond to mechanical or chemical factors [9].

Target sequences are the tripeptide motif Arg-Gly-Asp (RGD), triple-helical GFOGER peptides, two homologous sequences Leu-Asp-Val (LDV), and Ile-Asp-Ala-Pro (IDAP) [7,10]. RGD binds to α_v_β_1_, α_v_β_3_, α_v_β_5_, α_v_β_6_, α_v_β_8_, α_5_β_1_, α_8_β_1_, and αII_b_β_3_. RGD receptors are expressed by a wide range of cell types, including, for example, astrocytes, leukocytes, and fibroblasts [11]. GFOGER =0binding integrins are collagen receptors, including α_1_β_1_, α_2_β_1_, α_10_β_1_, and α_11_β_1_ integrins, which are expressed by fibroblasts, stem cells, and epithelial cells. The tripeptide LDV is recognized by the leukocyte receptors α_4_β_1_, α_4_β_7_, and β_2_ subunits, which are expressed mainly on leukocytes and epithelial cells [11] (Figure 1). The target sequence IDAP is, besides LDV and RGD, a peptide sequence apparent in fibronectin and is a recognition site for α_4_β_1_ [10].

This review aims to provide an overview of the role of αβ integrins on immune cells, with a focus on β_2_ integrins for the maintenance of immune homeostasis and the induction and course of immune responses, including infections, autoimmunity, and tumorigenesis. The importance of integrins on malignant cells for tumorigenesis has recently been well reviewed in [12]. The pathophysiological importance of β_2_ integrins is confirmed by the phenotype of patients lacking integrin activity who suffer from recurrent severe infections but are also prone to developing autoimmune diseases. Similar defects have been recapitulated in appropriate mouse models used to thoroughly delineate the immunological functions of β_2_ integrins in health and disease. In accordance, a number of agonists and antagonists of integrin receptors have been developed and evaluated for therapeutic applications.

## 2. αβ Integrin Immune Functions and Targeted Therapies

### 2.1. RGD-Binding Integrins

The RGD subfamily comprises a large group of integrins that interact with many different ligands, as reviewed in [13], including ECM components such as collagen, fibronectin, vitronectin, and tenascin [14] as well as surface receptors like E-cadherin. RGD receptors are essential for vascular smooth muscle cell migration, phagocytosis, and the activation of TGF-β, which regulates innate immunity and anti-inflammatory surveillance [11]. Most of the eight RGD-binding integrins are expressed in various tumor types, regulating pathophysiological processes like tumor invasion and metastasis formation [15]. However, they are also known to be involved in other diseases like sepsis [16] as a systemic response to infection that may be fatal due to organ failure [17], fibrosis [18] that is caused by inflammation-induced excessive release of fibronectin-forming fibrils to enable collagen deposition [19,20], and viral infections [21]. In this regard, integrin targeting is highly appealing for the delivery of therapeutics. For example, RGD-loaded exosomes were shown to yield therapeutic effects in breast cancer models after intravenous injections [13,22]. Furthermore, preclinical studies showed that RGD integrin antagonists, such as RGD-based cyclic peptides, efficiently suppressed tumor angiogenesis [23]. The most prominent example is cilengitide, an α_v_β_3_/α_v_β_5_ antagonist employed for the treatment of glioblastoma, which, however, failed its clinical endpoint [13]. Interestingly, the administration of low-dose cilengitide in vitro and in animal models revealed that it promoted tumor angiogenesis instead of inhibiting it [24]. So far, no successful RGD-targeted peptide therapy has been made available [13].

### 2.2. Laminin-Binding Integrins

Laminin receptors play critical roles in regulating cell adhesion, proliferation, migration, and survival, and they are important for organ development [11]. Similar to RGD-binding integrins, laminin receptor expression has also been shown to correlate with tumor progression [25]. Moreover, mutations in the receptor α_6_β_4_ resulted in junctional epidermolysis bullosa, a blistering skin disease [26]. So far, no specific therapy targeting laminin-binding integrins is available.

### 2.3. Collagen-Binding Integrins

The collagen receptor α_1_β_1_, also known as very late antigen 1 (VLA-1), was first identified in activated T cells [27]. VLA-1 acts as a promoter of inflammatory responses, and it facilitates monocyte transmigration by binding collagen XIII [11]. SAN-300 is a monoclonal antibody (mAb) directed against α_1_β_1_ integrin and was evaluated in a clinical phase II study for the treatment of patients with rheumatoid arthritis (RA) [11]. Furthermore, in animal disease models, inhibiting the interaction of α_1_β_1_, integrin, and collagen impaired T cell accumulation in the epidermis and consequently prevented psoriasis [28]. The integrin α_2_β_1_, also known as VLA-2, is expressed on platelets and mediates collagen binding at sites of inflammation. In this regard, vatelizumab, a mAb targeting the α_2_ subunit (CD49b) of VLA-2, was investigated in a clinical phase II study in multiple sclerosis (MS) patients [29]. After treatment with vatelizumab, higher frequencies of regulatory T cells (Treg) were observed in MS patients, which might have resulted from the inhibition of p38 mitogen-activated protein kinase (MAPK) signaling that is critically involved in the polarization of T helper (T_H_)17 cells and is activated by the α_2_ integrin cytoplasmic domain. Even though the clinical trial did not reach the end point, the blockade of VLA-2 may serve to shift the T_H_17/Treg polarization balance toward Treg [29].

### 2.4. Leukocyte Receptors

Leukocyte receptors play critical roles in leukocyte recruitment and (auto-)inflammatory diseases. The leukocyte receptor’s binding integrin subfamily comprises β_2_ integrins, which are discussed in more detail in the following section, and the integrins α_4_β_1_ (VLA-4; CD49d/CD29) and α_4_β_7_ [2]. The α_4_β_1_ integrin was shown to be necessary for leukocyte recruitment to the inflamed central nervous system (CNS) in experimental autoimmune encephalomyelitis (EAE), a murine MS model [30]. Its interaction with VCAM (vascular cell adhesion molecule)-1 mediated leukocyte adherence in the CNS. This observation prompted the development of Natalizumab, an IgG4 mAb directed against α_4_, which blocks α_4_β_1_ integrin and has been approved for MS treatment [31]. Hence, Natalizumab constitutes the first targeted therapy for the treatment of relapsing-remitting MS [2].

Furthermore, Vedolizumab and Etrolizumab also target α_4_β_7_ integrin [2,32]. Vedolizumab is a humanized IgG1 mAb that proved to be effective for the treatment of moderate-to-severe Crohn’s disease [2]. Etrolizumab is a humanized mAb that selectively binds the β_7_-subunit of α_4_β_7_ and α_E_β_7_ integrin heterodimers, thereby antagonizing α_4_β_7_/Mucosal address cell adhesion molecule (MAdCAM-)1-mediated lymphocyte recruitment and α_E_β_7_/E-cadherin interaction [33]. Etrolizumab yielded therapeutic effects in patients with severe inflammatory bowel disease (IBD) [34]. Moreover, Kunkel et al., using β_7_^−/−^ mice, demonstrated that α_4_β_7_ integrin was involved in the adhesion of leukocytes to Peyer’s patch endothelial venules [35]. Treatment with β_7_- and MAdCAM-1-specific mAb relieved experimental murine chronic colitis [36].

In addition, the α_4_β_1_ integrin was shown to play a critical role in hematopoietic stem cell (HSC) homing. Its binding to VCAM-1 caused HSC retention in the bone marrow (BM) [9]. The importance of α_4_β_1_ integrin for HSC biology has been confirmed by Rettig et al., showing that α_4_ integrin knockout mice presented with elevated numbers of HSC in the bloodstream compared to wild-type littermates [37]. In accordance, treatment of wild-type mice with the VCAM-1 antagonist Bortezomib increased HSC migration [38]. Therefore, Bortezomib is of great interest to enrich and isolate HSC from the blood of healthy patients for use in transplantations [9].

## 3. The β_2_ Integrin Family

### 3.1. Family Members and Their Ligands

**Figure 2:**
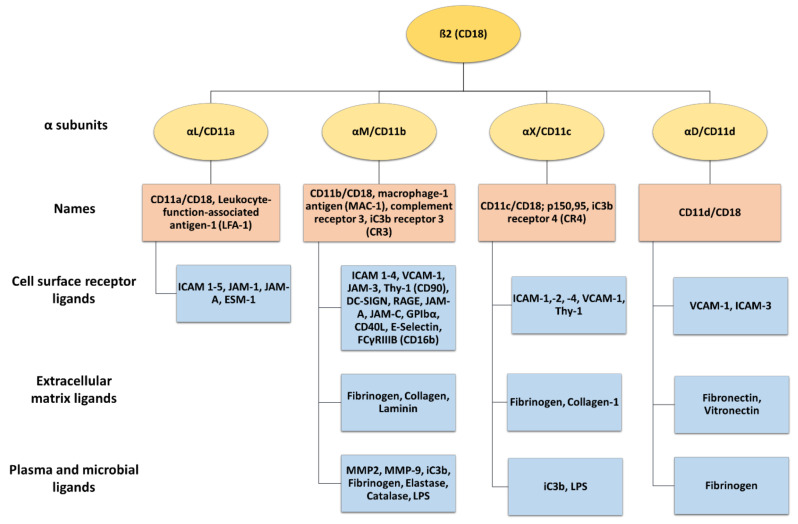
The β_2_ integrin receptor subfamily and their ligands. The common β_2_ integrin subunit (CD18) can pair with one of four α subunits (α_L_-CD11a, α_M_-CD11b, α_X_-CD11c, and α_D_-CD11d), forming LFA-1, MAC-1, complement receptor 4 (150.95/CR4), and CD18/CD11d, respectively. LFA-1 is the only β_2_ integrin expressed on T cells, while CD11b/CD18, CD11c/CD18, and CD11d/CD18 are also expressed on myeloid cells. β_2_ integrins can interact with a wide range of ligands, including cell surface receptors, extracellular matrix ligands, plasma, and microbial ligands. Created with BioRender.com.

β_2_ integrins belong to the leukocyte receptor subfamily of αβ integrins, and their expression is confined to leukocytes [3,8]. These heterodimeric surface receptors are composed of a variable α (CD11a-CD11d) and a common β_2_ (CD18) subunit, thereby forming CD11a/CD18 (α_L_β_2_, LFA-1), CD11b/CD18 (α_M_β_2_, MAC-1, complement receptor 3 (CR3)), CD11c/CD18 (α_X_β_2_, p150.95, and CR4), and CD11d/CD18 (α_D_β_2_) (Figure 2). β_2_ integrins commonly bind ligands via the αI domain [4], and the α subunit of each β_2_ integrin determines the extracellular ligand specificity to different ligands. These comprise a heterogenous group, including, for example, intracellular adhesion molecules (ICAM), VCAM, and endothelial cell-specific molecules (ESMs), as well as other cell surface and serum molecules [3,5,39].

LFA-1 is expressed by all types of leukocytes [40] and in platelets [41]. Activated LFA-1 engages ICAM 1–5, junctional adhesion molecule (JAM-)1, JAM-A, and ESM-1 [8,42,43]. Macrophage-1 antigen (MAC-1) is predominantly expressed by polymorphonuclear neutrophils (PMNs), monocytes, macrophages, and dendritic cells (DCs) [8,44]. MAC-1 binds many ligands, including surface receptors like ICAM 1–4, VCAM-1, JAM-3, receptor for advanced glycation end products (RAGE), thymus cell antigen 1 (Thy-1, CD90), platelet glycoprotein Ibα, DC-specific ICAM-3-grabbing non integrin (DC-SIGN) [8,45], E-selectin, and many others [46,47,48,49,50,51] (Figure 2). In addition, MAC-1 engages several matrix proteins like fibronectin and collagen [52,53], plasma proteins including fibrinogen, elastase, matrix metalloproteinase-2 (MMP-2), and MMP-9, and microbial ligands like lipopolysaccharide (LPS) [46]. Furthermore, MAC-1 is also known as a complement receptor 3 (CR3) and binds iC3b, facilitating the phagocytosis of complement-opsonized pathogens [54,55].

CD11c is a classical marker for murine DC, but in humans, it is also expressed by other myeloid cell types like monocytes/macrophages and PMN [56]. CD11c engages with diverse ligands such as ICAM-1, -2, and -4 [57,58,59,60], bacterial components like LPS [61], iC3b, as well as fibrinogen and collagen [62,63]. In addition, CD11c strongly binds VCAM-1, and as shown for monocytes, this serves to stop leukocyte rolling on endothelia, enabling transmigration through inflamed endothelial cells of the aorta [60].

CD11d/CD18 is the most recently discovered β_2_ integrin and is expressed under basal conditions by a few types of leukocytes, but it is most abundant in macrophages. In humans, CD11d/CD18 is highly expressed on natural killer (NK) cells, B cells, and γδ T cells [8,64]. It binds to cellular receptors like VCAM-1 and ICAM-3 [65], and, like MAC-1m to matrix proteins including fibronectin, vitronectin, and fibrinogen [4].

The expression of all β_2_ integrins is often upregulated in the case of cell activation [8], but as outlined in Section 3.2, their ligand affinity is controlled by signaling-dependent conformational changes.

### 3.2. Structure and Activation of β_2_ Integrins

**Figure 3:**
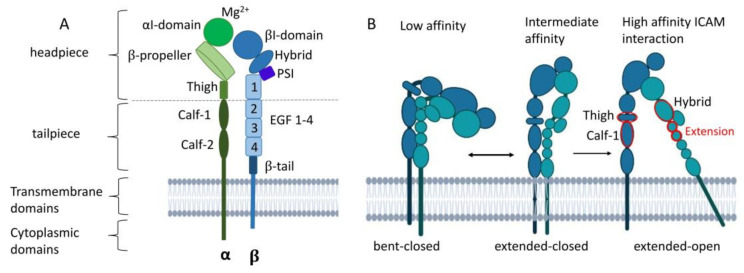
Structure of LFA-1 and conformational changes during integrin activation. (**A**) LFA-1 can be divided into three domains: an extracellular domain with a headpiece and tailpiece, a transmembrane, and cytoplasmic domain. (**B**) LFA-1 undergoes three conformational changes from bent-closed (low ICAM affinity) toward extended-closed with intermediate ICAM affinity state toward extended-open with high ICAM affinity. Upon ligand binding, the headpiece swings out of the hybrid domain with intermediate affinity. The extension between the thigh and calf-1 domain of the α subunit and the hybrid and EGF1–2 of the β domain (shown in red) results in the extended-one conformation with high ligand affinity. PSI, plexin–semaphorin–integrin. Created with BioRender.com.

The various α (CD11a–d) and the common β subunit (CD18) of β_2_ integrins are each composed of a long extracellular, a transmembrane, and a cytoplasmic domain [66] (Figure 3A). In the following section, the structure and mode of activation of β_2_ integrins are presented in more detail, referring often to LFA-1 as the common β_2_ integrin of all leukocyte populations.

#### 3.2.1. The α and β Subunit

The extracellular domain of the α subunit is comprised of a globular headpiece and a tailpiece. The former is composed of the αI unit, the β propeller, and the thigh domain. The lower tailpiece domain consists of the calf-1 and calf-2 regions [67,68]. By expanding the cleft between the β propeller and the αI domain, LFA-1 can interact with larger ligands. The top of the αI domain contains a metal ion-dependent adhesion site (MIDAS) that binds magnesium (Mg^2+^) to coordinate glutamic acid residues of ICAM-1 [69], which is required for conformational changes and thereby integrin activation [67,68].

The β subunit is connected to the cytoskeleton and transmits outside and inside signals [8]. The headpiece and the tailpiece of the β subunit consist of the βI domain, a so-called hybrid domain, a plexin–semaphorin–integrin (PSI) component, four epidermal growth factor (EGF)-like domains, and a β-tail. The βI domain also contains a MIDAS, which forms a bridge with a ligand or the glutamate residues of the αI domain [8,67,68].

#### 3.2.2. Activation of LFA-1

β_2_ integrins in the bent-closed conformation display interactions between the integrin headpiece and tailpiece and display only a low ligand affinity (Figure 3B). Upon ligand binding, LFA-1 undergoes sequential conformational changes, comprising straightening of the headpiece and tailpiece, yielding an extended-closed state as an intermediate. The tailpiece extends and opens the headpiece, with the hybrid domain swinging out. The extension movements of the ecto-domains occur between the thigh and calf-1 of the α subunit and between I-EGF1 and I-EGF2 of the β subunit. When the αI domain engages ICAM-1, a glutamic acid residue in the linker region becomes available to bind the βI MIDAS region [67,68]. Remodeling of the MIDAS within the βI domain and a ‘swing-out’ of the hybrid domain result in the extended-open state with a high ligand affinity [68,70,71]. Inside the cell, β_2_ integrin-binding adapter proteins like Talin-1 and Kindlin-3 that are attached to the cytoplasmic domain of CD18 cause the acquisition of the high-affinity state of β_2_ integrins [11]. At that state, β_2_ integrins can cluster and contribute to the formation of adhesion complexes. Integrin engagement and activation lead to bidirectional mechanotransduction and signaling across the plasma membrane of the cells.

#### 3.2.3. Inside-Out Signaling

**Figure 4:**
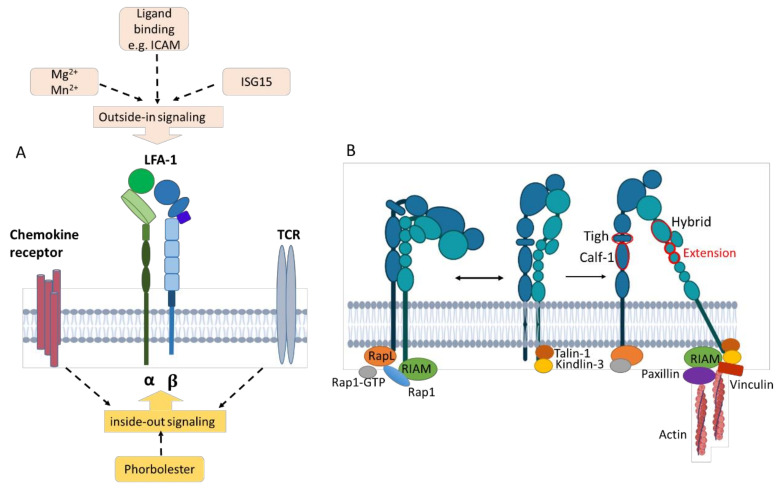
Triggering bidirectional inside-out and outside-in signals of LFA-1. (**A**) Inside-out signaling is triggered, e.g., by chemokine/cytokine signaling and TCR stimulation from within the cell, resulting in LFA-1 activation. Outside-in signaling is induced by ligand binding such as ICAM-1 as well as interferon-stimulated gene 15 and divalent cations (Mg^2+^, Mn^2+^). (**B**) Inside-out signaling through chemokine or TCR stimulation recruits Rap1-GTP. RAPL engages CD11a and activates Rap-1. RIAM engages Rap-1 and the β subunit. The β subunit binds talin-1 and kindlin-3, stabilizing the intermediate- and high-affinity states. Additional binding of paxillin and vinculin forms a frame for interaction with cytoskeletal elements. Outside-in signaling activates adapter proteins such as talin-1, which mediate downstream signaling and mechanotransduction. Created with BioRender.com.

The multistep activation process from bent-close to extended-open, including the process through which intracellular signals induce integrin activation that favors its extension, is termed ‘inside-out’ signaling [11,72]. Inside-out signaling is mediated, for example, by cytokine/chemokine stimulation and T cell receptor (TCR) activation, leading to downstream signaling and cytoskeletal rearrangements [73] (Figure 4A).

Inside-out triggering results in the recruitment of Rap1-GTP [8,72]. Rap-1 activation is mediated by the regulator of adhesion and polarization enriched in lymphocyte (RAPL) molecules that engage CD11a. RIAM (Rap-1-GTP interacting adaptor molecule) engages with both active Rap-1 and the integrin β subunit [74] and binds talin-1 and kindlin-3 [8,75], which stabilizes the intermediate and high-affinity receptor states by binding to different membrane motifs of the β subunit [11,75,76] (Figure 4B). Talin is the main intracellular binding protein that disrupts the interaction between the α and the β integrin subunits, thereby extending the tailpiece of the β subunit [77]. The interaction between talins and F-actin is important for anchoring LFA-1 to the actin cytoskeleton [76]. Additional binding of paxillin and vinculin forms a frame for interaction with cytoskeletal elements [75] since both constitute adaptor proteins with multiple binding sites for other adhesion components like focal adhesion kinase (FAK). FAK is a cytoplasmic tyrosine kinase that is activated by intramolecular interactions and, in turn, phosphorylates paxillin, promoting the exposure of additional protein docking sites that can regulate downstream processes such as migration and chemotaxis [11].

#### 3.2.4. Outside-In Signaling

The rearrangement of the cytoskeleton and intracellular responses due to β_2_ integrin binding to ligands induce outside-in signaling [78] (Figure 4A). This leads to the acquisition of a high-affinity state [73,79]. Engagement of ICAM and binding of soluble extracellular interferon-stimulated gene 15 (ISG15), an ubiquitin-like secreted protein, but also divalent cations such as Mg^2+^ promote outside-in signaling by binding the αI domain [75,80]. Both ISG15 [80] and LFA-1 cross-linking [81] have been reported to induce IFN-γ production. Outside-in signaling modulates gene expression, cell proliferation, survival, and apoptosis [11,82].

In response to activation, cytoskeletal proteins and kinases, as well as key adapters, assemble at the cell membrane, forming adhesion complexes that transfer signals from the ECM to the cell surface [11]. Adapter proteins in LFA-1-mediated signal transduction pathways can be differentiated as structural, scaffolding, and catalytic adaptors. Structural adaptors such as talin-1 recruit the protein complex Arp2/3 via association with vinculin, and this protein complex facilitates cytoskeletal reorganization. This so-called ‘molecular clutch’ is crucial for the mechanotransduction and activation of the cell [11,78]. Scaffolding adaptors like adhesion and degranulation-promoting adapter proteins couple LFA-1 and regulate F-actin clustering, cell polarization, and T-cell motility. Catalytic adaptors facilitate specific reactions like targeting protein kinase A and protein kinase C (PKC) to the membrane, cytoskeleton, and filopodia of migrating T cells [78].

### 3.3. Multifacetted Functions of β_2_ Integrins

Leukocytes are characterized by a cell-type-specific expression pattern of β_2_ integrins that may be altered, for example, in response to activation [8] and aging [83]. Whereas β_2_ integrins exert comparable functions in distinct cell types, like rolling on endothelial cells and phagocytosis of opsonized pathogens, they exert other functions in a more cell-type-restricted manner, such as, for example, controlling reactive oxygen species (ROS) production and the release of neutrophil extracellular traps (NETs). In the following section, general as well as more cell-type-specific properties of β_2_ integrins are discussed.

#### 3.3.1. Functions of β_2_ Integrins

##### Trafficking

Activated β_2_ integrins are crucial for the adhesion and trafficking of leukocytes from the bloodstream to sites of tissue inflammation by binding proteins of the ECM [5]. Leukocyte adhesion comprises multiple steps, including rolling along endothelia, adhesion, arrest, crawling, and finally, extravasation along a chemokine gradient to navigate to inflammatory sites [84]. Ligand binding to selectins and ICAMs induces β_2_ integrin clustering and the formation of multi-protein complexes that consist of intracellular signaling and adaptor proteins that connect β_2_ integrins with the cytoskeleton [85]. Leukocyte rolling is mediated by glycoproteins on the leukocyte cell surface, such as P-selectin glycoprotein ligand-1, which binds to endothelial selectins [9]. Chemotactic migration mediated by β_2_ integrins activates the p38 MAPK and phosphoinositide 3-kinase (PI3K) pathways. Chemokine stimulation enables β_2_ integrins to undergo a change in their conformation, thereby acquiring a high-affinity state. This leads to the binding of ICAMs on endothelial cells, resulting in a migration arrest [9,84,86]. LFA-1 and MAC-1 not only bind ICAM but also other surface receptors like RAGE and JAM receptors [45], and MAC-1 also engages CD90 [87], and all of these contacts contribute to trans-epithelial migration. Subsequently, integrin–ligand bonds are broken, reformed, and stabilized by cytoskeletal proteins such as talin and kindlin-3, which results in actin reorganization and spreading of the cells and, finally, leukocyte transmigration through the endothelium [3].

Regarding PMN migration, LFA-1 regulates adhesion to the endothelium, and MAC-1 facilitates intravascular crawling [88]. In the case of monocytes and lymphocytes, migration is mediated by LFA-1 [89]. Human monocytes, monocyte-derived macrophages, and monocyte-derived DC express both LFA-1 and MAC-1. Under physiological conditions, CD11c/CD18 was reported to be dominant, conferring adhesion to fibrinogen [56]. CD11d/CD18 and α_4_β_1_ were shown to be essential for leukocyte arrest during extravasation by binding to VCAM-1. Under steady-state conditions, CD11d/CD18 is not highly expressed and consequently might play a minor role in VCAM-1-mediated extravasation compared to α_4_β_1_ integrin. In contrast, under inflammatory conditions, CD11d/CD18 is highly expressed on human peripheral leukocytes and murine macrophage populations, which reflects its role in regulating, e.g., extravasation [4].

##### Phagocytosis

Phagocytosis is a unique form of cell endocytosis whereby cells internalize solid particles through vesicles, including microbial pathogens [90,91,92]. The complement receptors MAC-1 (CR3) and CD11c/CD18 (CR4) are essential for the phagocytosis of complement-opsonized pathogens by myeloid cell types via β_2_ integrin-induced Rho activity [3,93,94]. MAC-1 and CD11c/CD18 play important roles in the clearance of pathogens, cellular debris, and apoptotic cells opsonized with complementary factors, C3 and C4, respectively [8,95]. Moreover, apoptotic bodies derived from tumor cells can be taken up by MAC-1 on myeloid cells, NK cells, conventional (c)DC, and via CD36 by all DC populations, which in either case induces tolerance [96]. In addition, MAC-1 and CD11c/CD18 recognize iC3b-opsonized apoptotic cells, which attenuated pro-inflammatory cytokine production through nuclear factor kappa of activated B cells (NF-κB) inhibition [97].

##### Immunological Synapse

Besides their role in migration, LFA-1 and MAC-1 are also important constituents of the contact areas between antigen-presenting cells (APCs) and T cells required to confer T cell activation [98] and between immune effector cells and target cells to mediate cytotoxicity [99], generally termed the immunological synapse (IS). As previously shown by us, LFA-1 on CD4^+^ T cells is required for their polarization toward T_H_1 [100], but LFA-1 [101] and MAC-1 [102] on APC served as negative regulators of T cell activation.

##### Signaling

β_2_ integrins have been shown to regulate the responsiveness of leukocytes toward toll-like receptor (TLR) stimulation and have been implicated in various signaling processes [103], including, for example, calcium signaling [104], Src kinases [105,106], integrin-linked kinase [107], FAK [108], MAPK [109], Rho GTPases [110], and NF-κB [111].

#### 3.3.2. Cell-Type-Specific Expression Pattern and Activities

##### Innate Immune Cells

PMN

PMNs predominantly express LFA-1 and MAC-1 [8,112]. Heit et al. demonstrated that LFA-1 was important for chemotactic migration of PMN toward IL-8, whereas MAC-1 was required for chemoattraction toward N-Formyl-Met-Leu-Phe [113] and for transendothelial migration [114]. Moreover, sialyl Lewis x (sLe^x^), a known ligand of E-selectin [115], was reported to play a major role in PMN rolling [116]. In that study, Zen and coworkers demonstrated that MAC-1 and LFA-1 were decorated with sLe^x^-type glycans and recognized E-selectin. The removal of sLe^x^ completely interrupted Mac-1/E-selectin binding, and incubation with anti-sLe^x^ mAb significantly induced PMN degranulation, whereas transmigration through collagen-coated transwell filters and intestinal epithelial cell monolayers was inhibited.

Despite the pronounced role of LFA-1 and MAC-1 for PMN migration, we observed a larger infiltration of β_2_ integrin- [117] and MAC-1- [118] deficient PMN in mouse models of pulmonal aspergillosis, respectively, into the lung than that observed for the control animals. This observation underlined the fact that PMNs do not exclusively depend on β_2_ integrins for migration. In this regard, Mizgerd and coworkers identified VLA-4 as responsible for PMN infiltration into the lungs of CD18-deficient mice in acute bacterial pneumonia [119].

A key function of PMN is to kill pathogens intracellularly after MAC-1/CR3-conferred phagocytosis [120,121]. Moreover, MAC-1 physically interacts with FcγRIIA in human PMN by rearranging the cytoskeleton, enabling calcium-mediated signaling of Fc*γ*RIIA/B, resulting in phagocytosis and the release of pro-inflammatory cytokines [122]. In line with this, PMN of patients suffering from leukocyte adhesion deficiency (LAD)-1 due to impaired β_2_ integrin activity [123] (see Section 3.4.1) presented with impaired phagocytic activity that was mimicked by CD11b and CD18 blocking mAb, respectively [124].

Another anti-microbial mechanism of PMN is the release of granules, termed degranulation, of primary (containing pro-inflammatory and antimicrobial proteins) and secondary (e.g., NADPH oxidase and collagenase) granules into the phagosome, which prevents host tissue damage and kills pathogens [125]. Horie and Kita described human eosinophils involvement of MAC-1 during degranulation in vitro [126]. Hence, the mAb-mediated blockade of CD11b and CD18, respectively, inhibited eosinophil adhesion and degranulation, whereas the blockade of CD11a had no such effect.

Further, activated PMN may release NETs, which are composed of chromatin associated with antimicrobial proteins that bind pathogens [127]. This kind of regulated PMN cell death has been termed NETosis [128]. Behnen and coworkers demonstrated that the formation of immune complexes (IC) activated human PMN and consequently NET formation, mediated by FCγRIIIb as a binding partner of MAC-1 [129]. ICs are present in multiple organs and are composed of autoantibodies, which arise in the course of autoimmune diseases and infections [130]. Simultaneous mAb-mediated blockades of CD11b and CD18 decreased IC-induced NET release by PMN [129]. On the contrary, the blockade of CD11a had no effect on NET production, suggesting a unique role of MAC-1 in this regard. However, McDonald reported that NETosis required LFA-1-dependent PMN/platelet interactions [131]. The interruption of PMN/platelet interactions in mouse and human models decreased vascular NET formation. In addition, the blockade of CD11b resulted in complete inhibition of ROS-dependent NET release during cellular infection by *A. fumigatus* in vitro [132]. Likewise, *A. fumigatus*-induced NETosis required ROS generation by NOX2 (NADPH oxidase 2) as a downstream effector of MAC-1 since blockade of CD11b decreased NOX2 production and consequently NET release.

In agreement with the overall role of β_2_ integrins for the anti-microbial effector mechanisms of PMN [133,134], we demonstrated that a PMN-specific knockdown of β_2_ integrins yielded an aggravated course of invasive pulmonal aspergillosis in *A. fumigatus*-infected mice [117]. These mice presented with a higher fungal burden, despite a higher abundance of PMN in the pulmonary tissue and lower levels of pro-inflammatory cytokines, such as TNF-α. PMN with impaired β_2_ integrin expression also showed lower phagocytosis and NETosis capacities compared to their wild-type counterparts. These findings largely corroborate our previous findings in the same disease model using CD11b^−/−^ mice [118]. The congruent finding of elevated PMN numbers in LAD-1 patients [123] as well as CD18^−/−^ mice [135] and mice with PMN-restricted β_2_ integrin deficiency [117] may indicate a compensatory effect to counteract attenuated functional capacity. Altogether, it is conceivable that MAC-1 plays a dominant role in the main pathogen killing mechanisms of PMN that are crucial for the early control of infection.

##### Monocytes/Macrophages

Human monocytes were reported to express all β_2_ integrins, while mouse CD11c/CD18 was apparent only on alveolar macrophages [136], and the expression levels of CD11d/CD18 were low in all monocyte/macrophage populations [8]. Interestingly, LFA-1 was not expressed by murine microglia, which allowed us to distinguish between microglial and CNS-infiltrating immune cells [137].

LFA-1 strongly binds ICAM-3, which is upregulated in apoptotic cells [138], and silencing of LFA-1 in macrophages decreased the clearance of apoptotic cells [139]. Tang et al. showed that the stimulation of M1 macrophages with, e.g., IFN-γ, yielded stronger phagocytosis of tumor cells due to the elevated expression of LFA-1 and CD11c/CD18 [140]. Additionally, enhanced CD11c expression in macrophage-enriched tumors was found to correlate with elevated patient survival rates [140]. Yuan et al. demonstrated that macrophage-derived exosomes are densely decorated with LFA-1 and that their passage through the blood–brain barrier was increased upon brain inflammation due to upregulation of ICAM-1 in the inflamed tissue [141].

MAC-1 was shown to be crucial for the polarization of pro-inflammatory (M1) macrophages by inducing the expression of microRNA Let7a [142]. CD11b inhibition promoted macrophage polarization toward an anti-inflammatory (M2) state and accelerated tumor growth, while the activation of CD11b shifted polarization toward pro-inflammatory M1 macrophages, favoring tumor suppression in animal models of murine and human cancers.

In human monocytes and macrophages, CD11c/CD18 was shown to be more important than MAC-1 in mediating adhesion to fibrinogen [56]. Khalaji and coworkers reported that the adhesion of monocytes to collagen type I increased in the elderly due to the age-dependent elevated activation of CD11c/CD18 [143]. Further, CD11c^+^ macrophages were reported to be proangiogenic and to play an important role in experimental choroidal neovascularization, a destructive angiogenesis in age-related macular degeneration [144].

In vitro-polarized M1 macrophages were found to express much higher levels of CD11d than M2 macrophages [145]. High expression of CD11d enabled the migration of M1 macrophages to sites of inflammation, while moderate CD11d expression on M2 macrophages was important for migration to mesenchymal tissues [145]. Thus, the relative expression of both β_2_ integrins may determine their role in leukocyte extravasation [4,146]. On the contrary, Yakubenko and coworkers showed that elevated expression of CD11d/CD18 in macrophages increased their cell adhesiveness and inhibited migration in vitro [147]. These results were confirmed in vivo using an anti-CD11d blocking mAb.

mAb-mediated ligation of CD11b and CD11c on human monocytes, respectively, but not on CD11a, increased NF-κB activity, which in turn enhanced the expression of pro-inflammatory IL-8, macrophage inflammatory protein (MIP-)1α, and MIP-1β [111]. In line with this, MAC-1 clustering and activation initiated a gene program reflecting vascular inflammation [148]. In agreement, MAC-1 deficiency resulted in attenuated vessel wall inflammation after the induction of experimental angioplasty. Further, MAC-1 was demonstrated to trigger a toll/IL-1 receptor superfamily-like signaling cascade 1, which induced NF-κB activity. However, MAC-1/ICAM-1 interactions on human macrophages were reported to indirectly inhibit TLR signaling by promoting the expression of anti-inflammatory IL-10 [3,149,150], SOCS (suppressor of cytokine signaling) 3, and A20-binding inhibitor of NF-κB activation 3 [148].

Further, TLR stimulation activated PI3K, and RapL-mediated inside-out signaling activated β_2_ integrins [149,151]. In turn, integrin outside-in signaling activated Src/Syk, which resulted in the degradation of the signaling transducers MyD88 and TIR-domain-containing adapter-inducing IFN-β and thereby attenuated TLR signaling [149]. Further, Querrey and coworkers identified MAC-1 as a negative regulator of TLR4/MyD88 signaling in non-classical monocytes after lung transplantation in a murine model of primary graft dysfunction [152]. When lungs derived from CD11b^−/−^ mice were transplanted to recipient wild-type mice, lung injuries were aggravated due to increased production of chemokine (C-X-C motif) ligand 2, which promoted PMN chemoattraction into the lungs. Yee and Hamerman [153] reported that CD18^−/−^ murine BM-derived macrophages and DC were hypersensitive toward various TLR ligands through exacerbated production of pro-inflammatory cytokines like IL-12 and IL-6. These data show that in general, MAC-1 ligation generates a negative activation signal for monocytes and macrophages but also suggests an individual cell-specific role of β_2_ integrins regarding their effect on TLR-mediated stimulation, depending on the homeostatic versus inflammatory conditions.

##### DC

DCs, like all leukocytes, express LFA-1, but the expression of the other β_2_ integrin family members considerably differs between DC subtypes and species. In humans, MAC-1 and CD11c/CD18 are expressed on cDCs but are absent on pDCs [136]. In mice, CD11c/CD18 is expressed by all DC subtypes, and therefore, CD11c is often used as a pan DC marker [154]. MAC-1 is mainly expressed by murine cDC2 [155]. CD11d/CD18 is expressed at a low level by human and mouse DC [136].

In murine BM-derived DC, MAC-1 was shown to constitute a component of the receptor complex that regulates TLR4 internalization and has been identified as a positive regulator of TLR4-induced signaling [156]. In line with this, murine CD11b^−/−^ DC displayed attenuated activation via TLR4 upon LPS stimulation, which in turn affected their T cell stimulatory capacity [156]. The activation of β_2_ integrins was inhibited in DC by CD18-binding cytohesin-1 and cytohesin-1-interacting protein (CYTIP) [8,157]. Cytohesin-1 upregulated RhoA activity in DC, which is important for chemokine-induced conformational changes of β_2_ integrins and consequently their activation [158]. We and others demonstrated that CYTIP sequestered cytohesin-1 in the cytoplasm, thereby limiting its interaction with β_2_ integrins [101,159]. Several studies have shown that various viral pathogens like Herpes simplex virus type 1 [160] and cytomegalovirus (CMV) [161] conferred degradation of CYTIP in DC, resulting in higher LFA-1 activity and thereby stronger cell adhesion.

Recently, we showed that DCs derived from mice with a DC-restricted β_2_ integrin knockdown overexpressed stimulation-induced cytokines as a result of enhanced activity of STAT (signal transducer and activator of transcription)-1, -3, and -5, and they concomitantly impaired expression of SOCS proteins [162]. In contrast, a gene enrichment analysis of DC with a CD11c-specific deletion of β_2_ integrins demonstrated reduced expression of genes involved in inflammatory pathways, including TNF-α signaling and IFN-γ responses [162]. Further, positive regulatory genes of TLR-induced signaling, like WD repeat and FYVE domain containing 1 [163], were upregulated, whereas COMM domain containing 2, an inhibitor of NF-κB-dependent gene expression [164], was significantly downregulated. In autoimmune EAE, these CD18-deficient DCs limited the T_H_1/T_H_17 auto-inflammatory response due to impaired induction of T-bet-expressing T cells, resulting in a delayed and milder course of disease. Altogether, these results suggest that β_2_ integrins on DC are responsible for the induction of inflammatory responses [162].

We demonstrated that, within the IS between APC and T cells, LFA-1 on the T cell side was important for T cell activation and T_H_1 polarization [100]. Of note, we also reported that active LFA-1 [101] and MAC-1 [102] on murine DCs inhibited T cell activation. This inhibitory effect of MAC-1 on T cell proliferation was also shown for human monocyte-derived DC [165], and furthermore, MAC-1 on APC can suppress antigen presentation and T_H_17 differentiation, leading to immune tolerance [3,166]. Interestingly, Nording et al. demonstrated that MAC-1 on DC is crucial for DC/platelet interaction by binding to the platelet glycoprotein Ibα [167]. Furthermore, activated platelets upregulate MAC-1 on DC. Moreover, the engulfment of apoptotic material via interactions of CD36 on apoptotic bodies with MAC-1 on DC suppressed their APC activity [96].

##### NK Cells

NK cells express mainly LFA-1 and MAC-1, and in humans, they have also been reported to express CD11c/CD18 [168,169] and CD11d/CD18 at high levels, especially upon infection and inflammation [170,171].

In resting human NK cells, LFA-1 had a low ligand binding affinity that was stimulated by engagement of the NK cell receptors 2B4 and CD16 [172]. The binding of complement C3a, known for its tumor-promoting and immunosuppressive functions, by LFA-1 has been demonstrated to induce acquisition of the high-affinity conformation of LFA-1 and decrease the infiltration of NK cells into the tumor microenvironment in various murine tumor models [173]. LFA-1 was reported to be important for the adhesion of NK cells to tumor cells [174], causing LFA-1 accumulation at the IS [175]. Pariani et al. proposed a Golgi-dependent trafficking pathway involving A kinase anchoring protein 350 (AKAP350) as crucial for NK cell cytolytic activity [176]. Perez and coworkers suggested in an in vitro study that LFA-1 could regulate the degranulation of human NK cells [177]. They showed that CD3^−^CD8^+^CD56^+^ NK cells contained a high amount of cytolytic granules, including perforin and granzyme A. Stimulation of LFA-1 on NK cells as well as their coculture with target cells increased the release of perforin and, accordingly, the cytolytic activity requiring LFA-1/ICAM-2 interactions. Further, phospho-epitope profiling revealed that LFA-1 activity was dependent on p44/42 MAPK pathway activation. In line with this, the inhibition of p44/42 MAPK decreased LFA-1-directed perforin release and, hence, the cytolytic activity of NK cells. March and Loing reported that the stimulation of LFA-1 by incubation with plate-bound ICAM-1 polarized granules in a Syk-, phospholipase C-, PKC-, and paxillin-dependent manner [178].

MAC-1 has been defined as a marker for NK cell maturation in mice [179]. NK cells obtained from CD18-deficient mice showed hyporesponsive properties in vitro as well as alterations of their developmental program in vivo [180]. They also presented with somewhat diminished and missing self-recognition. However, the early immune response to mouse CMV infection was unaffected.

In agreement with the role of LFA-1 and MAC-1 for the cytolytic function of NK cells, β_2_ integrin deficiency largely abrogated their responsiveness toward stimulation [170]. However, the role of CD11c/CD18 and CD11d/CD18 in human NK cells has scarcely been analyzed so far.

##### Adaptive Immune Cells

B cells

B cells express mainly LFA-1 [181], but the expression of MAC-1 [182], CD11c/CD118 [183], and CD11d/CD18 [171] has also been observed in some subsets, as outlined in the following section. LFA-1 is necessary for the binding of B cells to ICAM-1 [184]. In human and mouse models, LFA-1 was found to be expressed at the highest level in memory B cells (MBCs), which consequently had a higher capacity to bind to ICAM-1 and VCAM-1 than naïve B cells [185].

The expression of MAC-1 was reported to be mainly restricted to CD27^+^ MBC and to contribute to migration, as evidenced by mAb-mediated blocking experiments [182]. Furthermore, MAC-1 attenuated B cell receptor (BCR) signaling to maintain autoreactive B cell tolerance and reduce TLR-3-dependent NK stimulation [8,186]. Further, B1 cells that differ from conventional B cells by producing polyreactive natural antibodies to counteract pathogens [187] have also been shown to express MAC-1 and were found to be enriched in systemic lupus erythematosus (SLE) patients [188]. Here, this B1 subpopulation expressed more CD86 and acted as an efficient APC. In CD11b^−/−^ mice, BCR stimulation resulted in stronger B cell proliferation, survival, and autoantibody production, as observed for wild-type B cells, suggesting a role of MAC-1 in maintaining B cell tolerance [186].

Rubtsov et al. reported on murine CD11b^+^CD11c^+^ B cells, termed age-associated B cells (ABCs), that accumulated in old female mice and in young SLE-prone mice of either gender [189]. ABCs were also observed in older female patients suffering from autoimmune diseases. Altogether, these findings suggested a direct role of CD11b^+^CD11c^+^ B cells in autoimmunity [190]. Nagy-Baló et al. showed that human tonsillar MBC expressed CD11c/CD18 to a low extent, which strongly increased upon BCR stimulation [191]. CD11c/CD18 is important for the proliferation, migration, and adhesion of BCR-activated MBCs to fibrinogen-covered follicular dendritic cells (FDCs). Such contacts facilitate the binding of antigens stored by FDCs, thereby promoting B cell survival and proliferation [192]. CD11c^+^ MCBs were characterized by the strong expression of genes involved in B cell activation, differentiation, and antigen presentation, thereby constituting precursors of antibody-secreting cells [183]. Rincon-Arevalo and coworkers observed higher frequencies of CD11c^+^ B cells in the blood of SLE patients than in samples of healthy donors. Interestingly, in either donor group, CD11c^+^ B cells showed higher expression levels of CD19, CD45RO, CD45RA, CD69, and Ki-67 and diminished expression of CD21 and C-X-C motif chemokine receptor 5 than CD11c^−^ B cells [193].

MAC-1 and CD11c/CD18 expression on B cells has also been observed in several B cell malignancies, such as chronic lymphocytic leukemia (CLL) [194]. Functional studies suggested a role for these receptors in the IL-10 production of CLL B cells in response to CpG stimulation [195]. Song et al. showed that CD11c-expressing B cells arise outside of germinal centers in cases of (auto-)inflammation and aging, depending on T follicular helper cells [196]. In cases of resolved infections, such B cells were apparent in the marginal zone of the spleen, depending on LFA-1 and VLA-4 activity, and constituted memory B cells readily activated to produce antibodies upon secondary infection. Human B cells also express CD11d, but its functional importance has not been thoroughly investigated [171].

##### T Cells

Of all β_2_ integrins, T cells express only LFA-1 [98]. In the case of APC/T cell interaction, LFA-1 is positioned in the peripheral supramolecular activation cluster (p-SMAC) of the IS. LFA-1 is linked to the intracellular proteins talin, kindlin-3, and Rap1, which stabilize the interaction between the TCR and major histocompatibility complex II (MHCII)-loaded peptide within the center of the (c-)SMAC [3,197,198]. On the T cell side, the c-SMAC is also enriched in TCR-associated co-receptors like CD3, CD4, and CD8 [8,199,200,201] as well as CD28, which engages costimulatory receptors (e.g., CD80, CD86) of the APC [202]. Upon activation and binding to ICAM-1, LFA-1 contributes to T cell activation and polarization of the T cell response [66,100].

**Figure 5:**
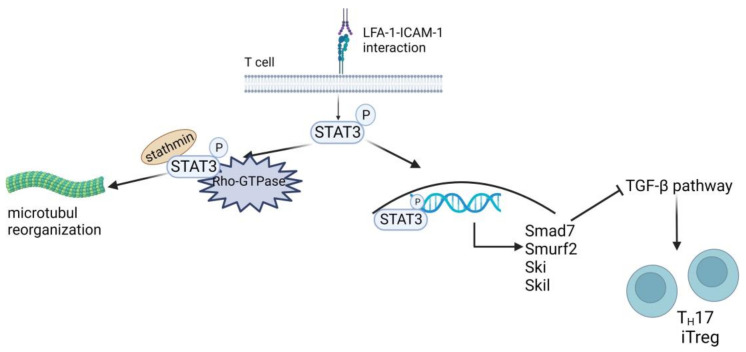
Role of LFA-1-mediated contact for T_H_17/iTreg polarization. LFA-1 triggers STAT3 activation and interrupts TGF-β signaling. LFA-1 triggered STAT3 activation by phosphorylation (P), translocating STAT3 to the nucleus, and its interaction with stathmin and Rho GTPase controls microtubule dynamics. In the nucleus, STAT3 upregulates the expression of the TGF-β-inhibiting transcription factors SMAD7, SMURF2, SKI, and SKIL. These proteins hinder TGF-β-mediated inhibition of IL-2 secretion and T-cell differentiation into T_H_17 or iTreg cells. Created with BioRender.com.

Normally, T cells engage APC via multiple short-term contacts called kinapses [203] before long-lasting contacts are formed to achieve their full activation. The conformational change in LFA-1 can lead to a 10,000-fold increase in affinity for ICAM-1 on APC [66,204]. Bleijs and coworkers observed that low-affinity binding of ICAM-3 in combination with high-affinity binding to ICAM-1 induced the formation of large LFA-1 clusters [205]. Moreover, low-affinity LFA-1/ICAM-3 interactions contributed to stabilize LFA-1/ICAM-1-dependent cell–cell contact. The high-affinity adhesiveness of LFA-1 proved essential for full T cell activation [75]. Moreover, the LFA-1/ICAM interaction influenced human T cell polarization by modulating T_H_1 and T_H_2 responses [73,206]. LFA-1 cross-linking also phosphorylated the signal transducer and activator of transcription 3 (STAT3), which is associated with tubulin-depolymerizing stathmin, thereby controlling cytoskeletal reorganization in migrating human T cells (Figure 5, left part) [207].

Tregs are a specialized subgroup of CD4^+^ T cells that serve to maintain self-tolerance by inhibiting the activation of autoreactive T cells, and they can be divided into naturally occurring thymus-derived Foxp3^+^ (t)Treg and peripheral (p)Treg [208,209]. The latter originates from extrathymic naive CD4^+^Foxp3^−^ T cells upon TCR stimulation in the presence of TGF-β [209,210] and upon antigen presentation in the absence of co-stimulation [211], respectively. LFA-1 has been shown to be required for Treg differentiation in vivo [212,213] and for Treg induction in vitro [214]. However, Verma and coworkers demonstrated that LFA-1-mediated activation of STAT3 induced expression of various genes associated with TGF-β pathway inhibition, including SMAD7 (mothers against decapentaplegic family member 7), SMURF2 (SMAD ubiquitination regulatory factor 2), SKI (Sloan-Kettering Institute proto-oncogene), and SKIL (SKI-like proto-oncogene) (Figure 5, right part) [206]. The induction of an LFA-1/ICAM-1 interaction-dependent refractory state in mouse and human CD4^+^ T cells toward TGF-β attenuated pTreg induction and polarization toward T_H_17 (Foxp3^+^RORγt^+^).

Tregs suppress T cell immune responses by various mechanisms, including anti-inflammatory cytokines (e.g., IL-10, TGF-β) [215,216], the depletion of IL-2 [217], the induction of apoptosis [218,219], metabolic pathways [220,221], and the modulation of the maturation and function of APC [215,222,223,224,225]. Concerning the latter, Tregs were reported to inhibit the upregulation of costimulatory receptors on DC via LFA-1 on the T cell side by binding ICAM on DC [226,227]. Further, Chen et al. demonstrated that strong LFA-1-dependent adhesions between Treg and DC led to cytoskeletal rearrangements in the latter, attenuating their ability to engage naïve T cells due to the Treg-focused displacement of cytoskeletal components [228]. In line with this, we observed a higher state of DC activation in mice with a Foxp3-specific knockdown of LFA-1 [229]. In accordance, in in vitro Treg/DC cocultures, LFA-1-deficient Treg interacted to a lower extent with DC, and the latter presented with a markedly activated immunophenotype. Moreover, LFA-1-deficient Tregs were characterized by the impaired expression of genes associated with suppressive activity, and the corresponding mice were characterized by systemic T_H_2-biased inflammation. Altogether, our findings suggested that LFA-1 was not only important for Treg induction but was essential for maintaining the tolerizing activity of Treg.

In addition to its role in T cell differentiation and activation, LFA-1 on activated CD8^+^ T cells is required to kill infected target cells by sealing the IS between the T cell and the target cell to direct cytolytic granules [3,66]. Furthermore, LFA-1 can function as a so-called mechanical gate to attract granules containing perforin and granzyme and trigger their fusion with the synaptic membrane [230]. Degranulation takes place mostly in regions of active force exertion within a cytotoxic synapse, suggesting that mechanosensing is involved in the release of cytolytic granules [230,231]. Similar to PMN, CD4+ and CD8+ T cells reportedly form T cell extracellular traps (TETs) [232], which are associated with skin diseases and leishmania infections [233]. It is unknown yet whether LFA-1 activation is involved in TET formation.

### 3.4. Leukocyte Adhesion Deficiencies

**Figure 6:**
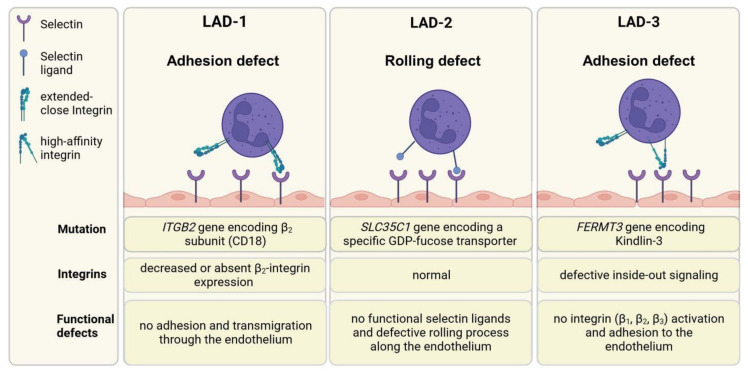
Leukocyte adhesion deficiency syndromes. The trafficking of leukocytes is a multistep process, including rolling along endothelia, adhesion, arrest, crawling, and finally, extravasation. LAD-1 syndrome is characterized by decreased or absent β2 integrin expression, resulting in impairment of leukocyte firm adhesion and migration into tissue. LAD-2 patients have a deficiency in selectin ligand expression, resulting in insufficient rolling of leukocytes. Humans with LAD-3 syndrome have no functional kindlin-3, which is an important adaptor protein for correct integrin activation. β1, β2, and β3 integrins cannot switch to their high-affinity state and interact with their ligands. Consequently, leukocytes are not able to adhere to the endothelium. Created with BioRender.com.

Leukocyte adhesion deficiencies (LADs) are rare hereditary disorders characterized by a defect in leukocyte trafficking. Decreased expression or activity of adhesion or adaptor proteins results in the manifestation of three different types of LADs, as summarized in Figure 6 [234].

#### 3.4.1. LAD-1

The crucial role of β_2_ integrins in the immune system is emphasized by the phenotype of LAD-1 patients, who suffer mainly from frequent and severe infections [235]. LAD-1 affects about one per million people worldwide [236]. In LAD-1 patients, a mutation in the CD18 gene leads to the attenuated expression of functional heterodimeric β_2_ integrins, resulting in impaired leukocyte adhesion and migration into the tissue [237] and attenuated pathogen-killing functions, e.g., of PMN [238]. In some patients, CD18 expression differs between cell types, and accordingly, the severity of LAD-1 can be subdivided into mild (>30% of patients), moderate (2–30%), and severe forms (<2%) [239]. About 75% of patients with severe LAD-1 die by the age of 2 years. Patients with moderate LAD-1 survive childhood, but their mortality amounts to 50% by the age of 40 years [240].

The most prevalent symptoms are recurrent bacterial infections of the skin, periodontitis, and poor wound healing [134,241,242]. In addition to these immunodeficiency symptoms, LAD-1 patients may suffer from autoimmune phenomena like IBD, type-1 diabetes, and nephritis [243,244]. In accordance with this, it has been demonstrated that LAD-1 patients with IBD presented with a higher number of Foxp3^+^ Tregs, but these displayed reduced suppressive activity [227,245]. Until now, the only curative therapy was allogeneic hematopoietic stem cell transplantation [246,247].

#### 3.4.2. LAD-2

LAD-2 results from a mutation in the *SLC35C1* gene that encodes a specific GDP-fucose transporter [248]. This causes a rolling defect of leukocytes by the aberrant expression of Sialyl Lewis X (sLeX) and other selectin ligands. Selectin ligands are carbohydrates that share many structural features with SLeX, whereby fucose is an essential element of all selectin ligands [249]. LAD-2 patients lack these fucosylated glycoconjugates and may show similar symptoms as LAD-1 patients, but may also present with severe mental and growth retardations [250]. The fucosylated glycoconjugates are synthesized by fucosyltransferases from the donor substrate, GDP-fucose. Thus, the defect in fucose metabolism can be partially compensated by oral fucose substitution therapy [251]. By this fucose is taken up into the cells, GDP-fucose is synthesized and transferred into the Golgi apparatus for utilization by fucosyltransferases. Therefore, the lack of selectin ligands can be compensated, normalizing PMN counts and function. Interruption of this therapy leads to the loss of selectin ligands after several days. Of note, the metabolic pathway that causes the psychomotor and growth retardations is still unknown.

#### 3.4.3. LAD-3

LAD-3 patients present with a mutation in the *FERMT3* gene encoding kindlin-3, which is important for integrin activation on leukocytes and thrombocytes [252,253]. Affected patients suffer from leukocytosis and recurrent infections resembling symptoms of LAD-1. Kindlin-3 is a cytoskeletal adaptor protein that interacts with the cytoplasmic tails of β_1_, β_2_, and β_3_ integrins, thereby shifting the receptors to a high-affinity state via inside-out signaling [254]. Hence, kindlin-3 deficiency precludes integrin activation. As shown in *FERMT3*^−/−^ mice, the adhesion of LFA-1 to kindlin-3 and conformational activation are impaired compared to wild-type mice [255]. Consequently, β_1_ and β_2_ integrin-mediated adhesion fail [254]. In addition, LAD-III patients have a bleeding tendency [256], as kindlin-3 is necessary to confer conformational activation of integrin α(IIb)β_3_ as a prerequisite to bind fibrinogen for platelet aggregation to initiate blood clotting [257]. Furthermore, osteoporosis-like bone defects, as observed in some LAD-3 patients [258], may result from integrin dysfunction of bone-resorbing osteoclasts, as observed in kindlin-3^−/−^ mice [259]. The finding that osteoclasts derived from mice with a specific deletion of either β_1_, β_2_ or β_3_ integrins displayed a milder phenotype than kindlin-3^−/−^ osteoclasts suggested that only combined loss of kindlin-3-dependent integrin activity resulted in the osteopetrosis phenotype. Similar to LAD-1, the only available therapy is allogeneic hematopoietic stem cell transplantation, accompanied by erythrocyte/platelet transfusions [260].

### 3.5. Role of β_2_ Integrins in Autoimmunity and Tumorigenesis and Receptor-Specific Therapeutic Approaches

As reflected by the impaired immune state of LAD-1 patients, immune cells critically depend on β_2_ integrins to eradicate pathogens but also to maintain immune homeostasis to prevent autoimmunity. Further, tumor-induced immunoregulatory cell types support tumorigenesis in a β_2_ integrin-dependent manner. Consequently, the suitability of β_2_ integrin agonists and antagonists for therapeutic applications has been assessed in a number of pre-clinical and clinical studies, as recently reviewed by Pang et al. [11].

#### 3.5.1. Autoimmunity

Autoimmune reactions are often characterized by a reduced number of functionally active Tregs that are critical to maintaining self-tolerance [261]. For example, reduced levels of Treg have been described in psoriatic arthritis [262], systemic SLE [263,264], and Kawasaki disease [261,265]. Further, we and others showed that LFA-1 was required for T_H_1/T_H_2 polarization [73,100,206]. An imbalance between T_H_1 and T_H_2 has been associated with several autoimmune diseases like MS, RA, and type-1 diabetes [266]. In this regard, LFA-1 signaling in combination with TCR stimulation triggered glycogen synthase kinase 3β-dependent Notch1 activation by γ-secretase proteolytic cleavage [267]. This upregulated T-bet expression and thereby favored a shift to T_H_1-associated cytokine production, specifically of IL-2 and IFN-γ. Notch constitutes a critical differentiation factor of T cell effector cells [268]. According to the so-called ‘second touch hypothesis’ [269], Notch1 signaling induced by LFA-1/ICAM interaction enhances T-cell effector priming and migration to sites of inflammation [73].

Furthermore, elevated expression of LFA-1 on T cells has been shown to correlate with systemic sclerosis and SLE, as well as RA and autoimmune thrombocytopenia [270]. mAb interfering with LFA-1/ICAM-1 interactions has been extensively analyzed in numerous pre-clinical studies [271]. Often, LFA-1 signaling interruption has been combined with additional therapies like the blockade of either ICAM-1 or CD40L. For example, in a preclinical study, combined treatment with anti-LFA-1 and -CD40L mAb after islet transplantation in vivo resulted in a tolerance that was defined as ‘dominant’, which was not achieved in the case of monotherapy with either murine mAb [272].

CD2 is known to upregulate LFA-1 avidity and, therefore, cell adhesion [273]. In a clinical phase II study, anti-LFA-1 and -CD2 mAb therapy in combination with T cell depletion prevented the rejection of transplanted bone marrow for the treatment of acute lymphoblastic leukemia in children [274]. However, this effect was not observed in adult patients [275].

Efalizumab, a humanized IgG1 anti-LFA-1 antibody, blocks LFA-1/ICAM-1 interactions in order to suppress T cell priming and effector functions of immune cells, which diminishes inflammatory cell recruitment and the reversal of keratinocyte hyperplasia at psoriatic lesions [276,277,278]. Despite this beneficial effect on skin lesions, efalizumab was not effective in psoriatic arthritis [279] and was less effective in psoriasis than other established therapies like cyclosporine and ultraviolet radiation-based therapies [280]. In 2009, efalizumab was withdrawn from the market because of a high risk of John Cunningham polyomavirus reactivation and the development of progressive multifocal leukoencephalopathy in patients under long-term administration [281,282,283]. In general, LFA-1-targeted therapies that aim to modulate the activation state of T cells carry the inherent problem that β_2_ integrins exert their function in a cell-specific manner and often have divergent functions on different cell types. For example, they limit T cell activation when expressed on DC [284], but they activate T cell functions when expressed on T cells [3].

MAC-1 was shown to suppress the differentiation of T_H_17 cells, which are associated with autoimmunity, and CD11b deficiency in mice was reported to cause elevated IL-6 production by APC, which in turn promoted T_H_17 differentiation [166]. In a murine collagen-induced arthritis model, CD11b^−/−^ mice presented with an earlier onset, higher incidence, and increased severity of arthritis compared to wild-type animals. This was due to markedly increased IL6 production by CD11b^−/−^ DC and elevated T_H_17 differentiation. In agreement, arthritis severity could be treated by the administration of IL-6-specific mAb treatments and the adoptive transfer of wild-type DCs [285]. Tang et al. investigated the role of MAC-1-FcγR interactions in immune complex-mediated injuries and compared the course of Fc-dependent anti-glomerular basement membrane nephritis in CD11b^−/−^ and wild-type mice. They found that initial glomerular PMN accumulation was comparable in both strains, but whereas it further increased in wild-type mice, PMN numbers decreased in CD11b-deficient mice. Furthermore, CD11b^−/−^ mice showed no proteinuria in contrast to wild-type controls [286]. In the context of the autoimmune disease bullous pemphigoid (BP), MAC-1 was shown to be important for disease development since CD11b^−/−^ mice were resistant to experimental BP, which was associated with less PMN infiltration into the skin [287]. Further, MAC-1 has been implicated in EAE induction, as CD11b-deficient mice displayed a significantly delayed onset and attenuated course of EAE [288]. We observed a similar outcome for mice with a DC-specific knockdown of β_2_ integrins [162]. β_2_ integrins on macrophages also play a significant role in the pathogenesis of osteoarthritis, and the current state of knowledge has recently been reviewed [289].

Several nonsynonymous single nucleotide polymorphisms in the *ITGAM* gene that encodes CD11b have been associated with an increased risk of developing SLE [290,291,292]. Cd11b agonists have been found to be promising candidates for the treatment of SLE [293]. Aside from SLE, these gene variants do not seem to be associated with an increased risk for other autoimmune diseases such as MS, type-1 diabetes, or Sjögren’s syndrome [294,295].

#### 3.5.2. Tumorigenesis

Tumors exploit several immune evasion mechanisms, including the induction and expansion of regulatory immune cell types comprising Tregs [296], tumor-associated macrophages (TAMs) [297], and myeloid-derived suppressor cells (MDSCs) [298]. The latter can be broadly defined as Ly-6C^+^CD11b^+^ monocytic MDSC and Ly-6G^+^CD11b^+^ granulocytic (g)MDSC that originate from monocyte and PMN progenitors, respectively [299]. PMN and gMDSC are scarcely distinguishable on the phenotypic level. Further, tumor-associated neutrophils (TANs) have been reported to exert tumoricidal (N1) and tumor-promoting (N2) properties within the tumor microenvironment (TME), respectively [300]. N1-TANs were reported to kill tumor cells via antibody-dependent cytotoxic effects (ADCC), like Fc receptor-dependent phagocytosis [301], which was shown to require MAC-1 [302,303]. Besides classical ADCC, Matlung and colleagues reported that N1-TAN engaged tumor cells opsonized by therapeutic antibodies via Fc receptors and MAC-1 [304]. Within the cytotoxic synapse, this resulted in disruption and internalization of part of the tumor cell membrane by N1-TAN, thereby inducing cell death by trogoptosis. Aside from ADCC, N1 TAN may kill Fas-expressing tumor cells via the Fas ligand [305], ROS [306], and nitric oxide [307]. Of note, MAC-1-dependent signaling plays an important role in both ROS [308,309] and nitric oxide [310] production.

TME-derived soluble mediators like G-CSF [311], IL-8 [312], and high mobility group box 1 [313] were demonstrated to promote NETosis. Whereas Schedel and coworkers reported that NET exerted anti-tumor effects by inducing tumor cell necrosis and inhibiting tumor cell motility [314], numerous other studies have reported on the tumor-promoting role of NETs via several mechanisms [315]. For example, Munir and coworkers demonstrated that cancer-associated fibroblasts (CAFs) released amyloid β peptide, which, presumably via MAC-mediated uptake, activated PMN to undergo NETosis [316]. NETs supported tumor metastasis by activating CAF [317], which suggests a positive feedback loop comprising CAF and PMN/gMDSC-derived NETs and promotes the binding of circulating tumor cells to liver sinusoids [318]. This in turn activated CAF, thereby promoting tumor growth.

N2-TANs have been reported to bind disseminated tumor cells via MAC-1/ICAM-1 and to facilitate their endothelial transmigration, thus favoring tumor metastasis [319]. Sprouse and coworkers demonstrated that interaction of N2-TAN/gMDSC with circulating tumor cells enhanced Notch1 receptor expression in the latter via ROS production [320]. This enhanced the engagement of JAGGED on N2-TAN/gMDSC, known to favor tumor cell proliferation [321], and elevated NODAL production and release by tumor cells [320]. NODAL, in a paracrine manner, activated N2-TAN/gMDSC by binding to the Cripto receptor.

Besides direct stimulation of tumor growth and metastasis, immunoregulatory cell types comprising N2-TAN, MDSC, and Treg exert immune-inhibitory effects by overlapping and distinctly soluble and receptor-dependent mechanisms both in the TME and in the periphery [322]. Aarts and coworkers reported that human MDSC required MAC-1-dependent cell–cell contact to suppress T cell proliferation, depending on ROS, degranulation, and trogoptosis [323]. As discussed above, all of these mechanisms were reported to rely in part on MAC-1 activity [126,132,304]. Likewise, as outlined above, Tregs require LFA-1 at least in part to exert their inhibitory effects on APC [226,228,229] and T cells [212,324]. Furthermore, MDSCs and Tregs have been shown to interact via soluble mediators and receptors, resulting in mutual activation, which may depend on β_2_ integrins as well [322].

CD11a^−/−^ mice were shown to display attenuated tumor growth, which was associated with lower Treg numbers [325]. In agreement, the treatment of wild-type mice bearing a subcutaneous tumor with a pharmacological LFA-1 antagonist (BIRT377) yielded similar results. Soloviev and coworkers reported that CD11b^−/−^ mice presented with attenuated melanoma growth due to impaired infiltration of the tumor by monocytes and PMN and reduced vascular endothelial growth factor production by the latter [326]. On the contrary, Schmid and coworkers showed that CD11b was necessary for the polarization of M1 macrophages, exerting tumoricidal activity by inducing the expression of miRNA Let7a, thereby attenuating tumor growth [142]. However, several studies showed that the application of CD11b-blocking antibodies attenuated tumor growth, especially when combined with radiation and immunostimulation by co-applied immune checkpoint inhibitors, respectively [327]. Pharmacological activation of MAC-1 using GB1275 (Leukadherin-1), which binds MAC-1 and activates this β_2_ integrin by outside-in signaling [328], yielded pronounced anti-tumor effects in numerous studies [142,329,330]. This outcome has been attributed largely to reduced tumor infiltration by myeloid cells and repolarization of MDSC toward an immunostimulatory phenotype, which was accompanied by elevated anti-tumor CD4^+^ and CD8^+^ T cell responses. In addition, Liu and coworkers demonstrated that CD11b agonists reprogramed murine and human TAM by inhibiting NF-κB activity and inducing stimulators of IFN genes and STAT signaling, resulting in IFN I production [331]. However, the oral MAC-1 agonist GB1275 failed to show therapeutic benefit in human patients when applied as monotherapy or in combination with immune checkpoint inhibition for the therapy of metastatic pancreatic adenocarcinoma in a clinical phase 1/2 trial (NCT04060342).

Altogether, these studies underscore the overall relevance of β_2_ integrins for tumorigeneses. However, data on their effect on tumor growth and modulation of the tumor microenvironment (TME) are still controversial, most likely due to the cell-type-specific, activation-dependent, and multi-facetted role of β_2_ integrins and especially MAC-1.

## 4. Conclusions

αβ integrins, and among these, β_2_ integrins, are highly relevant for immune functions by controlling intercellular communication, cell differentiation, activation, migration, and various effector mechanisms required to maintain self-tolerance and for pathogen killing. In line with this, patients lacking β_2_ integrin activity suffer from severe infections, and at the same time, they are prone to developing autoimmune diseases. All leukocytes express β_2_ integrins in a cell-type-specific pattern that is dynamically regulated in terms of expression intensity and ligand affinity in response to stimulatory events. So far, most studies on the cellular and molecular role of β_2_ integrins have been performed using mice with a constitutive knockout of either α (CD11a–d) or the common β (CD18) subunit. Therefore, it has been challenging to delineate the cell type-specific role of β_2_ integrins, especially in vivo, since functional alterations of immune cells may be due to intrinsic or extrinsic effects of integrin receptor deficiencies. To dissect our observations, we generated a mouse with a floxed CD18 gene, which enabled the establishment of sublines with a cell-type-specific knockdown of β_2_ integrins.

The modulation of integrin receptor activity by antibodies and pharmacological compounds has been shown to yield therapeutic effects in inflammatory diseases and for tumor therapy in preclinical studies, but in clinical trials, it quite often yields no major beneficial effects and is even associated with significant side effects. These outcomes are most likely a consequence of the systemic modulation of integrin activity. Hence, cell-type-directed delivery of integrin receptor-modulating agents by cell-type-targeting nanoformulations may be necessary to achieve a major breakthrough.

## Abbreviations

ABC	Age-associated B cell
ADCC	Antibody-dependent cytotoxic effects
ANA	Antinuclear antibody
APC	Antigen-presenting cell
BM	Bone marrow
BP	Bullous pemphigoid
CAF	Cancer-associated fibroblast
cAMP	Cyclic adenosine monophosphate
CCL	Chemokines like chemokine ligand
CLL	Chronic lymphocytic leukemia
CMV	Cytomegalovirus
CNS	Central nervous system
CR3	Complement receptor 3
c-SMAC	Central supramolecular activation cluster
CTLA-4	Cytotoxic T-lymphocyte-associated protein
CYTIP	Cytohesin-1-interacting protein
DC	Dendritic cell
EAE	Experimental autoimmune encephalomyelitits
ECM	Extracellular matrix
ESM	Endothelial cell-specific molecule
FAK	Focal adhesion kinase
FDC	Follicular dendritic cells
Foxp3	Forkhead box p3
gMDSC	Granulocytic myeloid-derived suppressor cell
HSC	Hematopoietic stem cell
IBD	Inflammatory bowel disease
IC	Immune complex
ICAM	Intercellular adhesion molecule
IFN	Interferon
Ig	Immune globulin
IL	Interleukin
IS	Immunolocial synapse
iTreg	Induced Treg
JAM	Junctional adhesion molecule
KO	Knockout
LAD-1	Leukocyte adhesion deficiency 1
LDV	Leu-Asp-Val
LFA-1	Leukocyte function-associated antigen 1
LPS	Lipopolysaccharide
mAb	Monoclonal antibody
MAC-1	Macrophage-1 antigen
MAPK	Mitogen-activated protein kinase
MCB	Memory B cell
MDSC	Myeloid-derived suppressor cell
MHC	Major histocompatibility complex
MIDAS	Metal ion-dependent adhesion site
MIP	Macrophage inflammatory protein
MS	Multiple sclerosis
NET	Neutrophil extracellular trap
NF-κB	Nuclear factor k-light-chain-enhancer of activated B cells
NK cells	Natural killer cells
NOX2	NADPH oxidase 2
PMN	Polymorphonuclear neutrophil
p-SMAC	Peripheral supramolecular activation cluster
pTreg	Peripheral Treg
RA	Rheumatoid arthritis
RGD	Arginylglycylaspartic acid
RIAM	Rap-1-GTP interacting adaptor molecule
ROS	Reactive oxygen species
SKI	Sloan-Kettering Institute proto-oncogene
SLE	Systemic lupus erythematodes
sLex	Sialyl Lewis x
SMAC	Supramolecular activation cluster
SMAD7	Mothers against decapentaplegic family member 7
SMURF2	SMAD Ubiquitination Regulatory Factor 2
SOCS	Suppressor of cytokine signaling
STAT	Signal transducer and activator of transcription
TAM	Tumor-associated macrophage
TCR	T cell receptor
Teff	Effector T cell
TET	T cell extracellular trap
TGF-ß	Transforming growth factor
TH	T helper cell
TLR	Toll-like receptor
TNF	Tumor necrosis factor
Tr1	Type 1 pTreg
Treg	Regulatory T cell
tTreg	Thymus-derived Treg
VCAM	Vascular cell adhesion protein
WT	Wild-type
